# High-Flow Nasal Oxygen in Patients with Acute Hypercapnic Respiratory Failure: A Narrative Review of the Physiological Rationale and Clinical Evidence

**DOI:** 10.3390/jcm13216350

**Published:** 2024-10-23

**Authors:** Gabriele Pintaudi, Salvatore Lucio Cutuli, Tommaso Rosà, Teresa Michi, Alessandro Cardu, Filippo Bongiovanni, Massimo Antonelli, Domenico Luca Grieco

**Affiliations:** 1 Dipartimento di Scienze dell’Emergenza, Anestesiologiche e della Rianimazione, Fondazione Policlinico Universitario Agostino Gemelli IRCCS, L.go A. Gemelli 8, 00168 Rome, Italy; 2 Dipartimento di Scienze Biotecnologiche di Base Cliniche Intensivologiche e Perioperatorie, Universita’ Cattolica del Sacro Cuore, Rome, L.go F. Vito 1, 00168 Rome, Italy

**Keywords:** respiratory failure, hypercapnic acidosis, critical care

## Abstract

Acute hypercapnic respiratory failure is a life-threatening condition caused by alveolar hypoventilation. It is mostly caused by an acute exacerbation of chronic obstructive pulmonary disease or conditions yielding muscle dysfunction. Noninvasive ventilation through a facemask is the cornerstone first-line strategy to support hypercapnic patients with acidemia, and current guidelines strongly recommend this intervention to improve survival and long-term clinical outcomes. Because of its benefits related to carbon dioxide washout from the upper airways and the enhanced comfort, high-flow nasal oxygen has been proposed as a respiratory support strategy in patients with hypercapnic respiratory failure, both as an alternative to and in combination with noninvasive ventilation. When compared to noninvasive ventilation as a first-line intervention, high-flow nasal oxygen shows a higher rate of failure. Hence, if not contraindicated, the use of noninvasive ventilation should be preferred. After the resolution of acidemia with noninvasive ventilation, high-flow nasal oxygen showed promising physiological effects compared to conventional oxygen. During weaning from mechanical ventilation in patients with or at risk of developing hypercapnia, high-flow nasal oxygen showed encouraging results, especially when applied alternating with sessions of noninvasive ventilation. Optimal settings of high-flow nasal oxygen in hypercapnic patients include the use of a smaller-size cannula, flows ranging between 30 and 40 L/min, and FiO_2_ adjusted to obtain SpO_2_ between 88% and 92%. Specific interfaces, such as asymmetric cannulas, may further enhance the benefits of a high flow in terms of carbon dioxide clearance. In this narrative review, we provide an updated overview of the physiological rationale and clinical evidence concerning the use of high-flow nasal oxygen in patients with acute hypercapnic respiratory failure.

## 1. Introduction

The biochemical hallmark of type 2 acute respiratory failure is hypercapnic acidosis (arterial carbon dioxide pressure [PaCO_2_] > 45 mmHg and pH < 7.35) [1]. This life-threatening clinical condition is caused by progressive carbon dioxide retention, due to both reduced alveolar ventilation and increased production from high metabolic demand [1].

Acute hypercapnic respiratory failure is frequently caused by an acute exacerbation of chronic obstructive pulmonary disease, whose pathophysiology involves inflammation of the upper airways (bronchi and bronchioles), eventually associated with bacterial or viral infections [2,3]. In this context, the inflammation of the upper airways may imply an increased expiratory flow resistance and dynamic hyperinflation due to bronchial secretions and bronchospasm, which increase lung volume and flatten the diaphragm, finally causing a reduction in its strength that yields alveolar hypoventilation; impaired gas exchange due to augmented dead space and an altered ventilation-to-perfusion ratio; and dyspnea due to respiratory muscle overload [4].

In current clinical practice, noninvasive ventilation providing inspiratory pressure support (and positive end-expiratory pressure) is the cornerstone of the initial management of acute hypercapnic respiratory failure; current guidelines strongly recommend this as a first-line intervention to support patients who develop hypercapnic acidosis [5]. Compared to invasive mechanical ventilation, noninvasive ventilation with a facemask showed comparable effects on gas exchange but was associated with a lower risk of ventilator-associated pneumonia, need for tracheostomy, shorter intensive care unit length of stay, reduced hospital re-admissions, improved survival, and long-term clinical outcomes [6,7,8]. Even when applied outside the intensive care unit, noninvasive ventilation was shown to improve hypercapnic acidosis and reduce endotracheal intubation and mortality rates [9]. However, the use of noninvasive ventilation applied with a facemask on carbon dioxide clearance may be limited by several factors influencing its appropriate delivery, such as poor patient tolerance to the interface (i.e., facemask, oro-nasal mask, and helmet), skin lesions (i.e., facemask and oro-nasal mask), patient–ventilator asynchronies, air leaks, and poor secretion clearance [10,11]. These become even more relevant when prolonged treatments are needed, so that the use of noninvasive ventilation is often limited to 6–12 h sessions, with frequent need for treatment interruptions.

In this context, high-flow nasal oxygen may represent an easy-to-use, attractive alternative noninvasive respiratory support strategy. High-flow nasal oxygen was initially proposed to support hypoxemic patients [12,13,14,15], but many of its physiological effects may translate into notable benefits for patients with acute hypercapnia and acidosis. However, the role of high-flow nasal oxygen in the management of patients with acute hypercapnia is still a matter of debate.

In this narrative review, we provide an updated overview of the physiological rationale and clinical evidence supporting the use of the high-flow nasal oxygen as a noninvasive respiratory support strategy in patients with acute hypercapnic respiratory failure.

## 2. Methods

This narrative review was based on a systematic search of PubMed for relevant English-language studies published from inception to August 2024. Study selection for our review included any observational studies, interventional trials, or reviews on adults with acute hypercapnic respiratory failure treated with high-flow nasal oxygen therapy.

We included studies describing (1) how to set up high-flow nasal oxygen therapy, (2) its physiological effects, and (3) clinical outcomes, with or without a comparison to other noninvasive respiratory support strategies.

Two independent reviewers performed an initial screening of all retrieved papers by title and abstract. Then, full-text screening was performed. At any stage, when discussion was unable to reach a definitive conclusion, disagreements were resolved by a third reviewer.

## 3. Physiological Rationale

**Figure 1:**
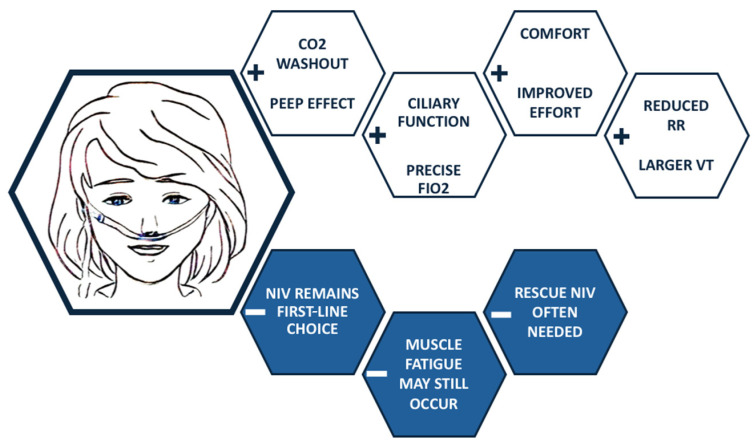
Pearls and pitfalls of high-flow nasal oxygen therapy in hypercapnic respiratory failure.

High-flow nasal oxygen delivers heated and humidified gas mixtures (up to 60 L per minute), at a prespecified fraction of inspired oxygen, through a symmetrical large bore nasal cannula [16,17]. This technique provides multiple physiological benefits in patients with acute hypoxemic respiratory failure of different aetiologies, for whom it has been demonstrated safe, feasible, and effective. In hypoxemic patients, high-flow nasal oxygen has been shown to reduce the need for endotracheal intubation in case of severe hypoxemic respiratory failure, without effects on mortality [18,19,20]. Accordingly, high-flow nasal oxygen is recommended as the first-line respiratory support strategy for this clinical condition [21,22]. In recent years, the use of high-flow nasal oxygen has also increased in the management of patients with acute hypercapnic respiratory failure. In these patients, high-flow nasal oxygen may confer several physiological benefits on gas exchange, airway patency, patient comfort, and work of breathing (Figure 1) [23,24].

High-flow nasal oxygen provides a high gas flow, which ensures the delivery of the set oxygen fraction with a limited dilution of inhaled gas [25]; it facilitates carbon dioxide washout from the anatomical dead space of the upper airways, which favours ventilatory efficiency and reduces the work of breathing. This effect is proportional to gas flow but reaches a plateau after 30–40 L per minute [13,26,27,28,29,30]. Moreover, the delivery of actively heated and fully humidified gas improves mucociliary clearance, moistens bronchial secretions, thus ameliorating cough effectiveness, and smooths the reactivity of inflamed airways to bronchospasm [31,32]. Furthermore, the high flows provide a flow-dependent positive end-expiratory pressure of 2–5 cmH_2_O, especially when the mouth is closed [15,33]. This prolongs expiratory time and may help counterbalance intrinsic positive end-expiratory pressure caused by dynamic hyperinflation due to altered elastic recoil of the lung and bronchospasm [34]. Finally, high-flow nasal oxygen is characterized by better tolerability and patient comfort compared to facemask noninvasive ventilation [18,24,35] among different patient populations, including those with acute hypercapnic respiratory failure due to chronic obstructive pulmonary disease exacerbation [36,37,38]. This finding is of utmost importance, as patient intolerance is one of the main factors limiting noninvasive ventilation use for prolonged periods and causing treatment failure. Intolerance is usually caused by claustrophobia and skin lesions, leading to air leaks and patient–ventilator asynchronies [11,39]. In this sense, high-flow nasal oxygen may represent an attractive alternative to noninvasive ventilation for treating hypercapnic patients. Available evidence, which is summarized in next paragraphs, indicates that high-flow nasal oxygen should not be preferred over noninvasive ventilation as a first-line intervention in hypercapnic patients, but may play a crucial role in combination with it, especially after hypercapnia resolution. During weaning from invasive mechanical ventilation in patients with or at risk of hypercapnia, high-flow nasal oxygen has shown encouraging results, especially when applied alternating with sessions of noninvasive ventilation. Optimal settings of high-flow nasal oxygen in hypercapnic patients include an inhaled oxygen fraction adjusted to obtain SpO_2_ between 88% and 92% and the use of flows ranging between 30 and 40 L per minute. Flows exceeding 40 L per minute may not be needed and could even be contraindicated, because they do not confer additional advantages on carbon dioxide clearance but may be associated with worsening hyperinflation due to excessive increases in expiratory resistance. Carbon dioxide washout may be further enhanced by cannulas having smaller or asymmetric diameters [40,41,42], but whether the use of these particular interfaces yields clinically relevant benefits has yet to be established.

## 4. Clinical Evidence

### 4.1. High-Flow Nasal Oxygen as Initial Treatment of Acute Hypercapnic Respiratory Failure

In patients with an acute exacerbation of chronic pulmonary disease who are hypercapnic but non-acidotic, some observational evidence reported that high-flow oxygen improves gas exchange and work of breathing compared to conventional oxygen therapy [43,44], but randomized trials showed conflicting results [45,46]. A systematic review and meta-analysis of 39 studies (1864 patients from 17 randomized controlled trials, 439 patients from 14 crossover investigations, and 532 patients from 8 non-randomized studies) [47] showed no difference in the intubation rate (primary outcome) between patients treated with high-flow nasal oxygen and noninvasive ventilation. Moreover, the authors found no difference between these treatments in terms of oxygenation, carbon dioxide, and barotrauma, although high-flow nasal oxygen was associated with better patient comfort compared to noninvasive ventilation. The comparison between high-flow and conventional oxygen showed that patients who received high-flow nasal oxygen had a lower risk of treatment failure and improved carbon dioxide clearance, in the absence of different hospital length of stay and hospital readmission after three months [47]. In this setting, however, a secondary analysis of a randomized trial conducted [48] on 330 patients with an acute exacerbation of chronic obstructive pulmonary disease with high bicarbonate levels (i.e., indicating metabolic compensation of chronic hypercapnia) reported a prolonged hospital length of stay in patients who received high-flow nasal oxygen compared to conventional oxygen. This finding was justified by a lower escalation to noninvasive ventilation in patients who received high-flow nasal oxygen, thus potentially delaying this therapy with consequent worse clinical outcomes. Importantly, there are no validated tools to predict and monitor the risk of failure in patients with hypercapnic respiratory failure undergoing high-flow nasal oxygen. Taken together, similar to the evidence available for noninvasive ventilation [21], these data do not support the routine use of high-flow nasal oxygen to prevent disease progression in patients with acute exacerbation of chronic obstructive pulmonary disease who are not acidotic or do not display signs of respiratory distress.

In patients with an acute exacerbation of chronic obstructive pulmonary disease who are hypercapnic and acidotic, noninvasive ventilation is strongly recommended as first-line intervention [5]. However, observational studies [49,50] and small clinical trials [35,37,51] investigated whether high-flow nasal oxygen may exert comparable physiological and clinical effects and found no difference in the rate of endotracheal intubation between these strategies. Cong et al. [52] randomized 168 patients to receive either high-flow nasal oxygen or noninvasive ventilation and found that both strategies were comparable in terms of gas exchange improvement, although patients who received high-flow nasal oxygen experienced fewer complications and better comfort in comparison to those who received noninvasive ventilation [52]. Likewise, a subgroup analysis of a randomized controlled trial found similar effects of high-flow nasal oxygen and noninvasive ventilation on gas exchange, treatment failure, and intensive care unit and hospital length of stay [53]. In this context, Cortegiani et al. [54] randomized 80 patients with hypercapnia and mild-to-moderate acidosis to receive either high-flow nasal oxygen or noninvasive ventilation as initial respiratory support strategy and found that high-flow nasal oxygen was non-inferior to noninvasive ventilation in terms of arterial carbon dioxide 2 h after treatment start (Table 1). Nevertheless, escalation to noninvasive ventilation was needed in approximately one-third of patients randomized to the high-flow group due to persistent acidosis or signs of respiratory distress within 6 h after randomization, highlighting the benefits of noninvasive ventilation in this population. Finally, a systematic review and meta-analysis of 528 patients with acute hypercapnic respiratory failure included in eight randomized trials reported no differences in mortality, gas exchange, endotracheal intubation rate, and intensive care unit and hospital length of stay between patients treated with high-flow nasal oxygen or noninvasive ventilation [55]. However, the heterogeneity of the studies and the small number of patients limited the ability to pool the results and draw firm conclusions on this topic. Overall, it remains undemonstrated whether high-flow nasal cannula can be used instead of noninvasive ventilation for the initial management of patients with acute hypercapnic respiratory failure, and current guidelines do not recommend this therapy as a valid alternative to noninvasive ventilation in this setting [22]. Therefore, noninvasive ventilation remains the cornerstone intervention to revert hypercapnia in patients with acidosis. High-flow nasal cannula could be attempted in patients failing noninvasive ventilation because of discomfort, although it requires a close monitoring of gas exchange and vital signs. Indeed, in the acute phase of hypercapnia, high-flow nasal oxygen can be safely and effectively used between sessions of noninvasive ventilation or after hypercapnia resolution. In this context, it may confer physiological advantages in comparison to conventional oxygen [38]; whether these translate into clinically relevant benefits remains to be established.

### 4.2. High-Flow Nasal Oxygen as Post-Extubation Respiratory Support Strategy in Patients with or at Risk of Hypercapnia

Failure of weaning from mechanical ventilation is defined as the need for reintubation within 2–7 days after extubation. This condition occurs in about 10–20% of patients who are extubated after a successful spontaneous breathing trial, is burdened by prolonged mechanical ventilation and a longer duration of intensive care unit length of stay, and is independently associated with increased mortality [57,58,59]. From a pathophysiological standpoint, extubation failure is caused by unresolved or de novo acute respiratory failure, difficult management of bronchial secretions, ventilatory muscle dysfunction, congestive heart failure, and/or neurological impairment. In this context, an early recognition of patients who are at high risk of extubation failure and the delivery of optimal treatments are of paramount importance to limit the occurrence of this event. Several factors have been identified as predictors of extubation failure. The main clinical factors that have been identified to increase the risk of extubation failure include age above 65 years, congestive heart failure, moderate to severe chronic obstructive pulmonary disease, high disease severity scores, body mass index above 30 kg/m^2^, increased bronchial secretion, difficult weaning (more than two spontaneous breathing trials before extubation), two or more comorbidities, and duration of mechanical ventilation over 7 days [60].

In the last decade, several randomized trials investigated the effectiveness of high-flow nasal oxygen versus conventional oxygen or noninvasive ventilation after extubation, with the aim of limiting the need for reintubation and improving other clinical outcomes, such as gas exchange, pulmonary complications, and mortality [43,61,62,63,64,65]. All these studies focused on pre-emptive interventions applied immediately after extubation, while evidence regarding the use of curative strategies when acute respiratory failure develops significantly after extubation is poor [66,67]. The current guidelines of the European Respiratory Society [22] suggest the use of noninvasive ventilation over high-flow nasal oxygen in non-surgical patients at high risk of respiratory complications (conditional recommendation with moderate certainty of evidence). However, in non-surgical patients at low risk of complications, the use of high-flow nasal oxygen is suggested over conventional oxygen therapy (conditional recommendation with low certainty of evidence).

More recently, Whang et al. [68] conducted a systematic review and meta-analysis on 1457 patients at high risk of extubation failure from 13 randomized trials and found that high-flow nasal cannula did not affect reintubation and mortality in comparison to noninvasive ventilation.

In chronic obstructive pulmonary disease patients intubated because of acute exacerbation with hypercapnic acidosis, high-flow nasal oxygen was demonstrated to be non-inferior to noninvasive ventilation in terms of gas exchange and extubation failure [56,69,70]. However, in mechanically ventilated patients at very high risk of extubation failure, as hypercapnic patients usually are, continuous treatment with noninvasive ventilation was demonstrated to reduce the intubation rate and hospital length of stay compared to high-flow nasal oxygen alone [71]. Similarly, another randomized trial showed that, in patients at high risk of extubation failure, pre-emptive noninvasive ventilation alternating with high-flow nasal oxygen best optimizes weaning outcome in comparison to high-flow nasal oxygen alone, with the most benefit observed in patients who are overweight or obese [57,72].

Altogether, given the robust evidence supporting the use of noninvasive ventilation to favour weaning success in patients with or at risk of hypercapnia, or, more generally, those exhibiting the criteria that define a high risk of extubation failure, noninvasive ventilation, eventually alternating with high-flow nasal oxygen, should be preferred over high-flow nasal oxygen alone [22].

## 5. Future Perspectives

Recent findings shed light on possible techniques to enhance high-flow nasal oxygen efficacy in terms of carbon dioxide washout. Compared to larger-size cannulas, cannulas of a smaller size better facilitate carbon dioxide washout (but limit the positive end-expiratory pressure effect) and could hence be preferred in these patients. Moreover, the application of innovative asymmetrical prongs in patients with hypoxemic respiratory failure was demonstrated to improve carbon dioxide clearance and reduce minute ventilation and work of breathing compared to classical symmetrical cannulas [73]. This physiological effect was mediated by the sealing of the larger bore to the nostril, which generates higher positive airway pressure [40] than conventional cannulas. Consequently, a more pronounced pressure gradient between the nasopharyngeal tract and the nostril hosting the smaller bore [74] mitigates the turbulent gas flow observed with conventional cannulas, leading to better carbon dioxide washout. Clinically, asymmetrical cannulas were reported to mitigate the harmful effects of high respiratory rates and low gas flow (below 60 L per minute) on the efficacy of carbon dioxide clearance with conventional high-flow nasal cannulas [40].

In this context, a randomized crossover trial in hypercapnic patients with chronic obstructive pulmonary disease exacerbation is currently ongoing (NCT05829083) and will assess the effect of high-flow oxygen delivered by symmetric vs. asymmetric nasal cannulas on arterial carbon dioxide change at 3 h.

## 6. Conclusions

In recent years, the role of high-flow nasal oxygen in the management of hypercapnic respiratory failure has been increasingly explored. Even if there is a solid physiologic rationale supporting the use of high-flow nasal oxygen in patients with hypercapnic respiratory failure, clinical studies showed that the effects are milder than those of noninvasive ventilation. When compared to noninvasive ventilation as a first-line intervention, high-flow nasal oxygen showed a higher rate of failure; if not contraindicated, the use of noninvasive ventilation should, therefore, be preferred. Conversely, after hypercapnia resolution with noninvasive ventilation, high-flow nasal oxygen showed promising physiological effects compared to conventional oxygen. During weaning from mechanical ventilation in patients with or at risk of hypercapnia, high-flow nasal oxygen showed encouraging results, especially when applied alternating with sessions of noninvasive ventilation.

Optimal settings of high-flow nasal oxygen in hypercapnic patients include the use of smaller or asymmetric cannulas, with flows ranging between 30 and 40 L/min, and FiO_2_ adjusted to obtain SpO_2_ between 88% and 92%.

## Figures and Tables

**Table 1:** Main randomized clinical trials in patients with hypercapnic respiratory failure.

Authors, Year of Publication	Sites	Study Duration	Number of Patients	Inclusion Criteria	Intervention	Main Results
Sklar et al., *Ann. Intensive Care* 2018, [35]	Single centre, Canada	2015–2017	15	Adults (age > 18 years) with cystic fibrosis	HFNO or NIV for 30 min	In adult CF patients stabilized after indication for ventilatory support, HFNO and NIV have similar effects on diaphragmatic work per breath, but high-flow therapy confers additional physiological benefits by decreasing respiratory rate and minute ventilation
Papachatzakis et al., *Int. J. Environ. Res. Public Health* 2020, [37]	Single centre, Greece	2017–2018	40	Adults admitted to the Emergency Department for hypercapnic respiratory failure (PaCO_2_ ≥ 45 mmHg)	Randomization (1:1) into intervention group A (HFNO) vs. control group B (NIV)	HFNO showed similar in-hospital severe complications, length of stay, and mortality compared with NIV
Longhini et al., *Crit. Care Med.* 2019, [38]	Two centres, Italy	2015–2017	30	Adults receiving NIV for more than 24 h; pH ≥ 7.35 during NIV; Respiratory rate ≤30 breaths/min	Patients underwent five 30 min trials, with the first, third, and fifth trials using noninvasive ventilation, whereas the second and fourth were randomly conducted with either standard oxygen or high-flow oxygen therapy	During NIV interruption, PaCO_2_ and diaphragm displacement remained unchanged regardless of the modality of oxygen administration. Convectional oxygen therapy resulted in a remarkable increase of diaphragm thickening fraction
Xia et al., *Crit. Care* 2022, [46]	Sixteen centres, China	2017–2020	330	Adults with AECOPD and mild hypercapnia (pH ≥ 7.35 and PaCO_2_ > 45 mmHg) at admission	Patients were randomly assigned to either HFNO or conventional oxygen therapy	HFNO compared to conventional oxygen therapy did not reduce the need for intubation among acute COPD exacerbation patients with mild hypercapnia
Cortegiani et al., *Crit. Care* 2020, [54]	Nine centres, Italy	2018–2020	79	Adults with mild-to-moderate AECOPD (arterial pH 7.25–7.35 and PaCO_2_ ≥ 55 mmHg before ventilator support)	Patients were randomly assigned to either HFNO or NIV	HFNO was non-inferior to NIV as initial ventilatory support strategy in decreasing PaCO_2_ after 2 h of treatment
Tan et al., *Crit. Care* 2020, [56]	Two centres, China; randomized	2019–2020	96	COPD patients (age ≤ 85 years) with hypercapnic respiratory failure caused by broncho-pulmonary infection	Patients were randomly assigned either to the HFNO group or the NIV group after pulmonary infection control was achieved	Among COPD patients with severe hypercapnic respiratory failure who received invasive ventilation, the use of HFNO after extubation did not result in increased rates of treatment failure compared to NIV

Abbreviations: acute exacerbation of chronic obstructive pulmonary disease, AECOPD; high-flow nasal oxygen, HFNO; noninvasive ventilation, NIV; arterial partial carbon dioxide pressure, PaCO_2._

## Data Availability

Not applicable.
